# Correlation of T2 signal intensity of uterine fibroids with semiquantitative perfusion MR parameters in candidates for MR-HIFU ablation: analysis according to fibroid types

**DOI:** 10.1186/2050-5736-3-S1-O97

**Published:** 2015-06-30

**Authors:** Young-sun Kim, Hyo Keun Lim, Hyunchul Rhim

**Affiliations:** 1 Samsung Medical Center, Seoul, Republic of Korea

## Background/introduction

T2 signal intensity and perfusion MR findings are known to be important influencing factors for therapeutic response of uterine fibroids to MR-guided high-intensity focused ultrasound (MR-HIFU) ablation. While T2 signal intensity of fibroids is easy to assess, perfusion MRI is relatively difficult to perform. The aim of this study was to evaluate relationships between T2 signal intensity and semiquantitative perfusion MR parameters of uterine fibroids to know whether T2-weighted image can replace a role of perfusion MRI for procedure screening.

## Methods

A total of 170 most symptom-relevant, non-degenerated uterine fibroids (mean diameter, 7.3cm; range 3.0–17.2cm) in 170 women (mean age, 43.5 years, range 24-56) who underwent screening MRI exams for MR-HIFU ablation were retrospectively analyzed. Signal intensity of uterine fibroid was assessed as a ratio of T2 signal intensity of uterine fibroids to that of skeletal muscle. Parameters of semiquantitative perfusion MRI (100 dynamics, 3s time resolution) which included peak enhancement, relative peak enhancement (%; 0% refers same signal intensity as in precontrast image), time to peak (s), wash-in rate (/s), and wash-out rate (/s) based on analyses of time-signal intensity curve were investigated to know their relationships with T2 signal intensity ratio using multiple linear regression test. The correlations between T2 signal intensity and the independently significant semiquantitative perfusion MR parameter were then evaluated using Spearman’s correlation test according to the fibroid types.

## Results and conclusions

Multiple linear regression test revealed that only relative peak enhancement had an independently significant relationship with T2 signal intensity of uterine fibroid (B=0.023, p<0.001). Based on analyses according to fibroid types, submucosal protruding type failed to show significant correlation (n=20, rho=0.275, p=0.240) while significant correlations were noticed in all the other types (submucosal type, n=45, rho=0.629, p<0.001; intramural type, n=25, rho=0.411, p=0.041; transmural type, n=40, rho=0.493, p=0.001; subserosal type, n=40, rho=0.486, p=0.001).

Conclusion: In candidates of MR-HIFU ablation therapy for uterine fibroids, T2 signal intensity of non-degenerated uterine fibroid had an independently significant positive correlation with relative peak enhancement of semiquantitative perfusion MRI in most cases, other than in submucosal protruding type.

